# Experience-Driven Axon Retraction in the Pharmacologically Inactivated Visual Cortex Does Not Require Synaptic Transmission

**DOI:** 10.1371/journal.pone.0004193

**Published:** 2009-01-14

**Authors:** Kana Watanabe, Yu Morishima, Masahito Toigawa, Yoshio Hata

**Affiliations:** Division of Integrative Bioscience, Institute of Regenerative Medicine and Biofunction, Tottori University Graduate School of Medical Sciences, Yonago, Japan; Lund University, Sweden

## Abstract

**Background:**

Experience during early postnatal development plays an important role in the refinement of specific neural connections in the brain. In the mammalian visual system, altered visual experiences induce plastic adaptation of visual cortical responses and guide rearrangements of afferent axons from the lateral geniculate nucleus. Previous studies using visual deprivation demonstrated that the afferents serving an open eye significantly retract when cortical neurons are pharmacologically inhibited by applying a γ-aminobutyric acid type A receptor agonist, muscimol, whereas those serving a deprived eye are rescued from retraction, suggesting that presynaptic activity can lead to the retraction of geniculocortical axons in the absence of postsynaptic activity. Because muscimol application suppresses the spike activity of cortical neurons leaving transmitter release intact at geniculocortical synapses, local synaptic interaction may underlie the retraction of active axons in the inhibited cortex.

**Method and Findings:**

New studies reported here determined whether experience-driven axon retraction can occur in the visual cortex inactivated by blocking synaptic inputs. We inactivated the primary visual cortex of kittens by suppressing synaptic transmission with cortical injections of botulinum neurotoxin type E, which cleaves a synaptic protein, SNAP-25, and blocks transmitter release, and examined the geniculocortical axon morphology in the animals with normal vision and those deprived of vision binocularly. We found that afferent axons in the animals with normal vision showed a significant retraction in the inactivated cortex, as similarly observed in the muscimol-treated cortex, whereas the axons in the binocularly deprived animals were preserved.

**Conclusions:**

Therefore, the experience-driven axon retraction in the inactivated cortex can proceed in the absence of synaptic transmission. These results suggest that presynaptic mechanisms play an important role in the experience-driven refinement of geniculocortical axons.

## Introduction

Experience during early postnatal development plays an important role in the development of brain function and the refinement of specific neural connections. In the mammalian visual system, monocular visual deprivation (MD) in early life induces a loss of cortical response to a deprived eye together with a significant retraction of afferent axons from the lateral geniculate nucleus (LGN) serving the deprived eye (ocular dominance plasticity) [1]–[4]. Previous studies showed that activity-dependent mechanisms should play a crucial role in experience-driven plasticity. For example, MD does not induce ocular dominance plasticity when the activity of cortical cells and their inputs from LGN are both blocked by tetrodotoxin [5]. Moreover, previous works have demonstrated a role of the activity of cortical cells themselves in controlling the direction of ocular dominance plasticity. When cortical cells are selectively inhibited pharmacologically by a γ-aminobutyric acid type A (GABAA) receptor agonist, muscimol, leaving the activity of geniculocortical afferents intact, the open eye becomes less effective than the deprived eye [6]–[8]. Furthermore, active axons serving the open eye selectively retract and their cortical territory shrink, whereas axons from the deprived eye remain mostly intact [7], [9]. Subsequent experiments revealed that presynaptic activity in the absence of postsynaptic activity, which is induced by visually evoked activity in afferent axons and cortical inhibition, plays an important role in the retraction of open eye afferents in the inhibited cortex [10].

These findings suggest a crucial role of the activity of presynaptic axons in the determination of whether the retraction can occur. In previous experiments, afferent axons might have retracted by a presynaptic mechanism in response to, for example, depletion of a possible trophic signal from cortical cells, which was induced by inhibiting cortical cells. Alternatively, the signal for retraction might be delivered only to active afferents as a result of a local synaptic interaction with cortical neurons, because muscimol treatment inhibits cortical neurons but does not prevent synaptic transmission itself. For example, it was reported that insufficient activation of cortical neurons can induce the depression of synaptic transmission [11], which may lead to afferent retraction through a retrograde message. To determine whether local synaptic interaction underlies the axon retraction in the inhibited cortex, it would be useful to examine the effect of cortical inactivation by blocking synaptic inputs instead of applying a GABAA receptor agonist.

Botulinum neurotoxins are clostridial proteases that enter nerve terminals and act on the soluble N-ethylmaleimide–sensitive factor attachment protein receptor (SNARE) proteins. Botulinum neurotoxin type E (BoNT/E) specifically cleaves synaptosomal-associated protein-25 kDa (SNAP-25) and blocks transmitter release [12], [13]. Previous experiments demonstrated that BoNT/E injections can produce a long-term inactivation of the visual cortex and hippocampus by blocking spontaneous and evoked neuronal activity [14], [15]. Here, we inactivated the primary visual cortex of kittens by blocking synaptic transmission with cortical BoNT/E injections and examined the geniculocortical axon morphology in the animals with normal vision and those deprived of vision binocularly. We found that the afferent axons in the animals with normal vision show a significant retraction in the inactivated cortex, as similarly observed in the muscimol-treated cortex, whereas the axons in the binocularly deprived (BD) animals were mostly preserved. Therefore, the experience-driven axon retraction in the inactivated cortex can proceed in the absence of synaptic interaction.

## Results

### Functional effects of BoNT/E injection on visual cortex

**Figure 1:**
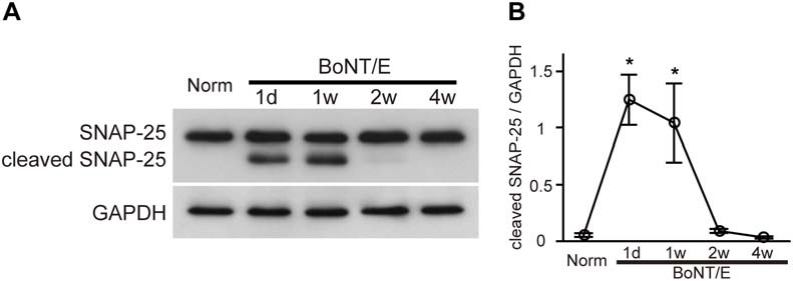
Cleavage of SNAP-25 by BoNT/E injection in visual cortex. (A) Representative immunoblots for SNAP-25 in rat visual cortex on various days after BoNT/E injection. Cleaved SNAP-25 with a lower molecular weight is clearly observed 1 day and 1 week after BoNT/E injection (upper panel). Norm, normal animals. (B) Quantification of cleaved SNAP-25. Blot densities were normalized to those of GAPDH. Error bars indicate SEM. *: P<0.05 (n = 3–5 each, one-way ANOVA followed by post hoc Scheffe test).

We first examined the effect of cortical BoNT/E injection on SNAP-25 and neuronal activity in the rat visual cortex. To estimate the duration of BoNT/E effects, we analyzed the time course of SNAP-25 cleavage after a single BoNT/E injection into the visual cortex. The visual cortical tissue was harvested on various days (1 day, 1 week, 2 weeks, and 4 weeks) after the injection of BoNT/E solution and subjected to western blot analysis using a monoclonal antibody that recognizes both intact and BoNT/E-cleaved SNAP-25. SNAP-25 cleavage was clearly detected 1 day and 1 week after the BoNT/E injection (Fig. 1). Cleaved SNAP-25 level decreased to undetectable level 2 and 4 weeks after the injection. Therefore, SNAP-25 cleavage in the visual cortex would persist for at least 1 week following BoNT/E injection. BoNT/E-injected animals did not show any sign of systemic intoxication during this time window.

**Figure 2:**
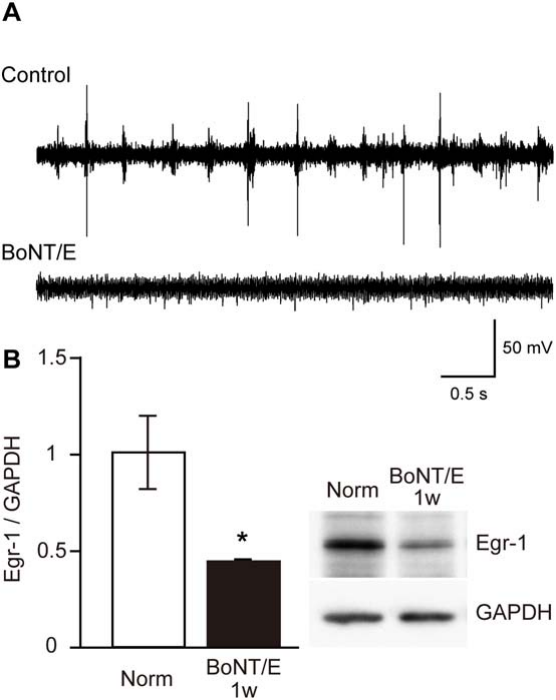
Effects of BoNT/E on cortical activity and Egr-1 expression level in visual cortex. (A) Examples of spike activity in rat visual cortex contralateral (upper trace, Control) and ipsilateral (lower trace, BoNT/E) to BoNT/E injection site recorded 1 week after injection. BoNT/E blocked neuronal activity only in the injected cortex. (B) Egr-1 expression level is significantly decreased in the BoNT/E injected cortex. The upper right panel shows examples of immunoblots for Egr-1 in the visual cortex of normal rats (Norm) and that 1 week after BoNT/E injection. Blot densities were normalized to those of GAPDH and expressed as the ratio to those of Egr-1 in normal animals. Error bars indicate SEM. *: P<0.05 (n = 5 each, unpaired t-test).

To determine the effect of BoNT/E injection on cortical activity, we recorded multiunit activity from the visual cortex of rats 1 week after BoNT/E injection. As shown in Fig. 2A, we did not observe any spike activity in the visual cortex treated with BoNT/E, whereas we found vigorous visual responses and spontaneous activity in the untreated visual cortex contralateral to the BoNT/E injection site. We further confirmed the blockade of neuronal activity in the BoNT/E-treated cortex by evaluating Egr-1 expression (Fig. 2B). The inducible expression of Egr-1 is commonly used as a marker of neuronal activity [16]. The level of Egr-1 expression significantly decreased in the BoNT/E-treated cortex compared with that in the cortex of normal animals (average±SEM: 43.5±1.2%, p<0.05, unpaired t-test). Thus, single BoNT/E treatment could effectively block neuronal activity in the visual cortex at least for 1 week.

### Effects of visual experience on arborization of geniculocortical axons in BoNT/E-treated cortex

**Figure 3:**
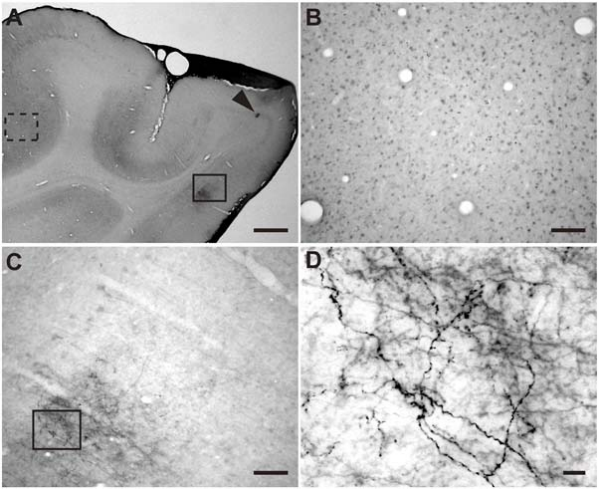
Example of labeled axons in BoNT/E-treated visual cortex. (A) Low-magnification image of coronal section across visual cortex of BoNT/E-injected kitten. An arrowhead indicates the BoNT/E injection site. (B, C) High-magnification view of two regions in A far from (dotted line, B) and close to (solid line, C) the injection site. Egr-1 signal is detectable in the area far from the injection site (B), whereas the signal is very low in the area near the injection site (C). The staining at the lower-left in C represents labeled axons. (D) Enlarged view of geniculocortical axons in BoNT/E inactivated area (box in C). Scale bar, A: 1 mm, B, C: 100 µm, D: 10 µm.

We labeled geniculocortical afferents of 6-week-old kittens by microinjecting an anterograde tracer, biotinylated dextran amine (BDA), into lamina A of LGN. Activity of the visual cortex was suppressed by disrupting synaptic transmission by cortical injection of BoNT/E. A group of kittens was deprived of vision in both eyes by eyelid suture. After 1 week, the cortical region inactivated by BoNT/E was delineated physiologically by mapping the activity of cortical cells. As similarly observed in rats (Fig. 2A), we found no spontaneous or visually evoked activity in the region around the BoNT/E injection sites. The inactivated area extended up to 10 mm in the anterior-posterior direction. We further confirmed the cortical inactivation by Egr-1 immunostaining (Fig. 3). Egr-1-immunopositive cells were distributed at a high density in the area far from the injection sites (Figs. 3A and B). In contrast, the density of Egr-1-immunopositive cells markedly decreased in the area near the injection sites (Figs. 3A and C). The region of very low Egr-1 immunoreactivity included the entire medial bank and often extended beyond the sulcus suprasplenialis and the sulcus lateralis. Cortical arbors of geniculocortical axons in the BoNT/E inactivated area were labeled well with BDA, as shown in Fig. 3D. We found no difference in the quality of labeling between the axons in BD animals and those in animals with normal vision.

**Figure 4:**
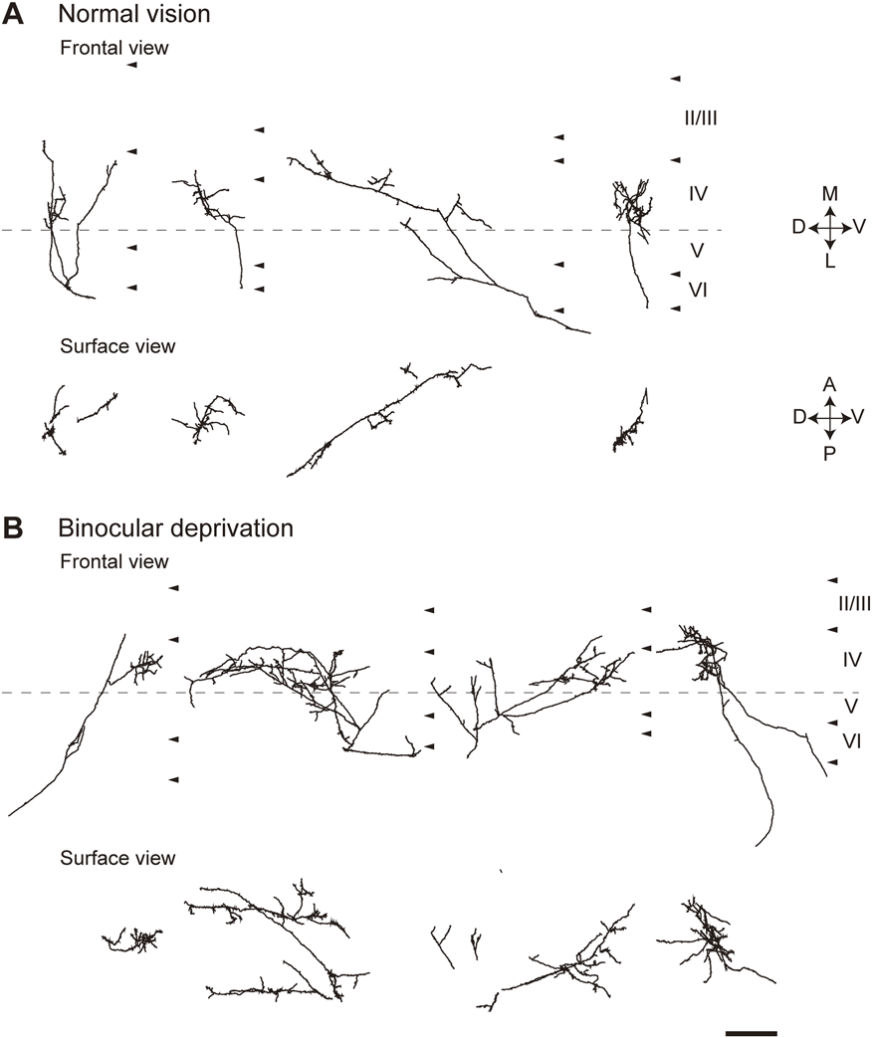
Examples of reconstructed arbors of normal-vision animals (A) and BD animals (B). Arrowheads on the right side of each arbor in frontal view indicate the borders between cortical layers. The arbors in frontal view are aligned on the border between layers IV and V. Surface view images were calculated on a computer by rotating frontal-view arbors 90° along the axis indicated by the dotted lines. Only portions above the layer IV and V border are shown as surface view images. The arbors in the inactivated visual cortex of normal-vision animals (A) appear to be smaller than those of BD animals (B). D, dorsal; V, ventral; M, medial; L, lateral; A, anterior; P, posterior. Scale bar, 400 µm.

In this study, 10 and 9 axonal arbors in the inactivated region of area 17 were reconstructed from the normal-vision animals (n = 5) and BD animals (n = 3) in three dimensions, respectively. Four examples of arbors from each group are shown in Fig. 4. All of the arbors in this study represent those serving the contralateral eye. The terminal arborizations of geniculocortical axons in both normal-vision and BD animals were mainly localized in layer IV. The arbors in the normal-vision animals showed a marked reduction in the complexity of terminal arborization compared with those in BD animals (Figs. 4A and B). In addition, the cortical area covered by each arbor in the normal-vision animals was much smaller than that in BD animals, as clearly shown in surface views of the arbors.

**Figure 5:**
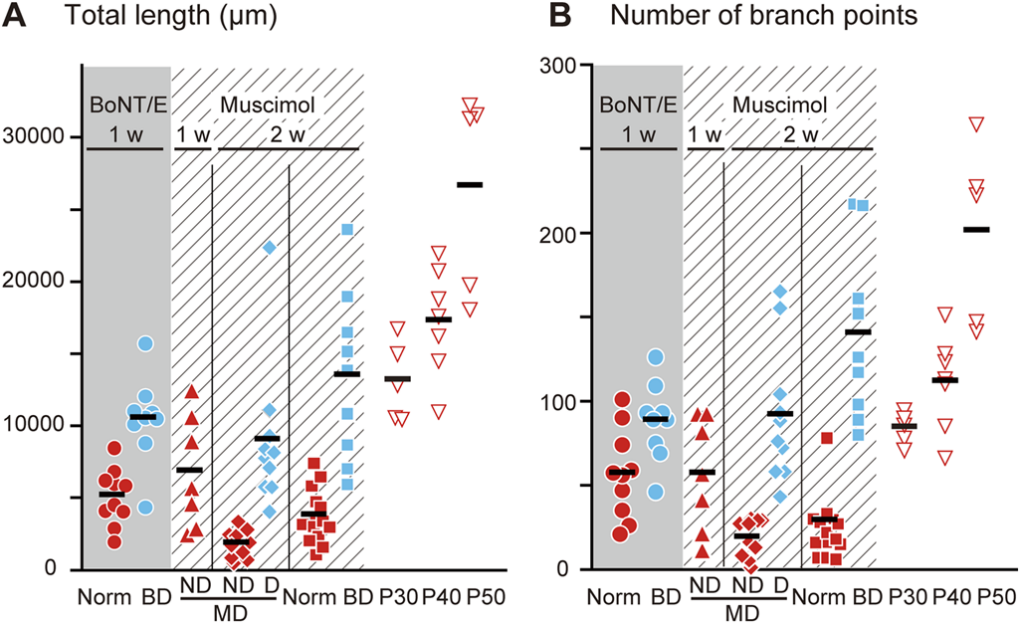
Quantitative analysis of arbor morphology. Scattergrams show a plot of two measures, total length (A) and number of branch points (B), for all arbors in this study together with the measures obtained under various experimental conditions published previously [9], [10], [17]. Total length (A) is calculated by adding the lengths of all axon segments constituting the terminal field of an arbor. The number of branch points (B) indicates the total number of axonal bifurcations. Only portions above the layer IV and V border are considered for these analyses. Each symbol represents the data of an arbor, and short horizontal lines indicate the means of individual groups. Red and blue symbols represent open-eye and deprived-eye arbors, respectively. The data obtained in the BoNT/E- and muscimol-treated cortex are indicated by the shaded and striped areas, respectively. The duration of cortical treatment is indicated at the top. Arbor and animal groups are indicated at the bottom as follows: Norm, arbors of animals with normal vision; BD, arbors of binocularly deprived animals; ND and D of MD, nondeprived-eye and deprived-eye arbors of monocularly deprived animals; P30, P40 and P50, arbors of normal animals on postnatal days 30, 40 and 50.

We quantitatively determined the axonal morphology by measuring two parameters, the total length of axon segments and the number of branch points of the cortical arbors of individual geniculocortical axons and compared them with published results for the arbors in the animals under various experimental conditions (Fig. 5 and Table 1) [9], [10], [17]. In the BoNT/E-treated cortex, the arbors in the normal-vision animals were significantly shorter and had fewer branch points than those in the BD animals. Small arbors in the normal-vision animals should reflect the net shrinkage of arbors rather than the suppression of development because they were significantly smaller than those at the start of BoNT/E injection (normal P40 animals). On the other hand, the arbors in the BoNT/E-treated cortex of the BD animals were preserved and similar in branching number to those in the normal animals at the start of BoNT/E injection (normal P40), although they were somewhat shorter in total length. Thus, in the cortex inactivated by BoNT/E injection, the arbors serving the open eye, which carry visual information, showed a highly significant shrinkage and the shrinkage could be alleviated by visual deprivation, as observed in the cortex inhibited by muscimol treatment [9], [10].

**Table 1:** Statistical comparison of geniculocortical arbors.

Cortical treatment	BoNT/E	Muscimol (1w)	Muscimol (2w)				None		
Arbor Groups	BD (P48–50)	MD-ND (P47–53)	MD-ND (P42–43)	MD-D (P41–45)	Norm (P40–46)	BD (P40–46)	P30	P40	P50
*Total length*									
BoNT/E-norm	0.001 ↓	ns	0.001 ↑	0.034↓	ns	0.001↓	0.002↓	0.001 ↓	0.002 ↓
BoNT/E-BD	-	ns	0.0001 ↑	ns	0.0001 ↑	ns	ns	0.004 ↓	0.003 ↓
*Number of branch points*									
BoNT/E-norm	0.027 ↓	ns	0.002 ↑	0.041↓	0.003 ↑	0.001↓	ns	0.003 ↓	0.002 ↓
BoNT/E-BD	-	ns	0.0001 ↑	ns	<0.0001 ↑	0.021↓	ns	ns	0.003↓

Statistical comparisons among arbor groups are provided as p values (Wilcoxon rank sum test). ns, not significant (p>0.05). Arrows for the p values indicate whether each of the two measures of the BoNT/E-norm and BoNT/E-BD groups shown on the left is larger (↑) or smaller (↓) than those of the arbor groups shown on the top. Arbor and animal groups: norm and BD, arbors of normal-vision and BD animals, respectively. MD-ND and MD-D, nondeprived-eye and deprived-eye arbors of MD animals, respectively. The ages of the animals in each group at perfusion are given in parentheses below each experimental condition.

The open-eye arbors in the muscimol-treated cortex (MD-ND and Norm in Muscimol 2w, Fig. 5 and Table 1) showed a much more significant shrinkage than those observed in the BoNT/E-treated cortex. The more significant shrinkage in previous muscimol experiments should reflect the longer cortical inactivation rather than a difference in pharmacological effects of BoNT/E and muscimol, because muscimol treatment for 1 week induced the same level of shrinkage as BoNT/E treatment (MD-ND in Muscimol 1w, Fig. 5 and Table 1, unpublished observation). On the other hand, the arbors in the BoNT/E-treated cortex of BD animals were similar to the arbors serving the deprived eye in the muscimol-treated MD animals, although they were shorter than those in muscimol-BD animals (MD-D and BD in Muscimol 2w, Fig. 5 and Table 1). Therefore, experience-dependent axon retraction can occur in the absence of synaptic transmission when cortical activity is suppressed.

## Discussion

In this study, the geniculocortical axons in the visual cortex inactivated by BoNT/E treatment showed a highly significant shrinkage and the shrinkage could be alleviated by visual deprivation (Figs. 4 and 5, Table 1), as observed previously in the muscimol-treated cortex [9], [10]. These results indicate that the axon retraction in the inactivated cortex can proceed in the absence of synaptic transmission.

BoNT/E is a metalloprotease that selectively cleaves the synaptic protein SNAP-25 and prevents transmitter release [12], [13]. Because administration of BoNT/E can block synaptic activity reversibly, it is used as a tool to address the role of synaptic activity *in vivo* [14], [15]. We found no spontaneous or visually evoked activity in the BoNT/E-treated region of the visual cortex even 1 week after the administration. Consistent with previous reports [14], [15], we did not find a sign of general deleterious effect of BoNT/E on the cortical tissue. The activity blockade was well confined to the vicinity of the injection sites, which extended up to 10 mm in the anterior-posterior direction, and we observed vigorous visual responses outside the area and in the contralateral visual cortex. In addition, we detected small activities in the middle cortical depth, possibly representing afferent activity, as similarly observed in the muscimol-treated cortex [9], [10], [18], indicating that BoNT/E did not disturb the electric activity of afferent axons. The Nissl-stained sections of the BoNT/E-treated cortex showed no differences in neuronal density and cortical thickness from those of the normal cortex. Finally, if BoNT/E had exerted a deleterious effect on afferent axons, such effect should have caused a degradation of presynaptic afferents for both eyes. The present finding that the retraction of afferents in the BoNT/E-treated cortex depends on their own activity argues strongly against this possibility.

Previous results in the muscimol-treated cortex suggest that presynaptic activity in the absence of postsynaptic activity leads to the retraction of afferent axons, on the basis of the assumption that muscimol selectively suppresses the activity of postsynaptic cells and does not have direct presynaptic effects. Pharmacological studies showed that muscimol binds selectively to GABAA receptors, which are located only on postsynaptic sites [19], [20]. However, it was also reported that muscimol at a high concentration might act on GABAB receptors, which are located on geniculocortical axon terminals [21]. This finding raises a possibility that the retraction of open-eye axons observed in the muscimol-treated cortex might be induced by activation of presynaptic GABAB receptors rather than by cortical activity blockade. The present finding that open-eye afferents retract in the BoNT/E-treated cortex provides evidence against this possibility because BoNT/E can inactivate the visual cortex without activating GABAB receptors.

The afferent arbors in the BoNT/E-treated cortex of normal-vision animals showed a significant retraction, because they were smaller than those at the start of BoNT/E injection (arbors in the normal P40 animals). The open-eye arbors in the cortex treated with muscimol for 1 week showed a retraction extent comparable to that in the BoNT/E-treated cortex (MD-ND in Muscimol 1w, Fig. 5 and Table 1). Thus, it is reasonable to state that cortical inhibition by BoNT/E induces the retraction of afferent axons to the same extent as cortical inactivation by muscimol. On the other hand, visual deprivation prevented afferent arbors from retracting in the BoNT/E-treated cortex, as found previously in the muscimol-treated cortex [10]. However, the deprived-eye arbors in the BoNT/E-treated cortex were much shorter in total length and had fewer branch points than the arbors in the normal animals at the time of perfusion (P50, Fig. 5 and Table 1), suggesting that cortical inactivation prevented an axonal elongation during normal development. Furthermore, the deprived-eye arbors in the BoNT/E-treated cortex were shorter in length than the arbors in the normal animals at the time of BoNT/E injection (P40, Fig. 5 and Table 1). Thus, the deprived-eye arbors in the BoNT/E-treated cortex might not be completely rescued from retraction and become smaller than the normal arbors, although the extent of retraction is much smaller than that of the open-eye arbors in the inactivated cortex.

The previous findings in the muscimol-treated cortex suggest a mechanism of axon retraction depending on the activity of presynaptic afferents. Only active afferents might express structural changes in response to a repulsive signal, which may be a repulsive substance or a depletion of trophic substances in the inactivated cortex, for example. It is also possible, however, that the signal for retraction might be delivered only to active afferents as a result of local synaptic interaction with cortical neurons, because muscimol treatment does not prevent synaptic transmission itself. The present results obtained in the BoNT/E-treated cortex, in which synaptic transmission was prevented, strongly argue against the latter possibility and support presynaptic mechanisms of open-eye axon retraction. The activity of axons might directly affect intracellular signals leading to morphological changes. Active afferents might have greater Ca^2+^ influx at their terminals than deprived-eye afferents. The difference in Ca^2+^ concentration might regulate axon behavior, as demonstrated in the guidance of growth cones [22]. Alternatively, presynaptic activity might regulate the response of axons to a repulsive signal in the cortex. For example, recent experiments demonstrated that p75 neurotrophin receptors mediate axon retraction during development of sympathetic and olfactory neurons [23], [24]. It is of interest to note that p75 was observed to be regulated in an activity-dependent manner in a study using neuronal cultures [25]. It is possible that such an activity-dependent regulation of repulsive interactions underlie the experience-driven axon retraction in the visual system.

Although such hypothetical presynaptic mechanism of axon retraction should operate also in the normal (active) cortex, the afferent axons serving the open eye of MD animals and those serving either eye in the normal-vision animals without muscimol infusion had much longer length and more branching than those in the inactivated cortex. When the cortex is active, cortical neurons may release some substance to rescue the axonal retraction or stabilize afferent synapses from the retraction. For example, neurotrophic factors are known to be released from target neurons and promote axonal growth [26], [27]. Especially, previous studies demonstrated that brain-derived neurotrophic factor (BDNF) influence the cortical projection of LGN axons (ocular dominance columns) [28], [29] and its expression is regulated in activity-dependent manner [30]. Therefore, two modes of plasticity which allow weakening and strengthening of particular pathways might operate concertedly in the developing visual cortex.

In conclusion, the present findings provide anatomical evidence that the experience-driven axon retraction in the inactivated visual cortex can proceed in the absence of synaptic transmission. These results suggest that presynaptic mechanisms play an important role in the experience-dependent rearrangement of LGN axons.

## Materials and Methods

All kittens (n = 8) in this study were born in the breeding colony of the Tottori University Research Center for Bioscience and Technology. All Long-Evans rats (n = 19) in this study were obtained from Shimizu Laboratory Animal Supply Co. Ltd (Kyoto, Japan). The experimental procedures used met the regulation of the animal care committee of the Tottori University.

### Surgery

A 30 G stainless steel cannula connected to a 10 µl Hamilton syringe with a polyethylene tubing was inserted into the left hemisphere of area 17 of the visual cortex in 6-week-old kittens (postnatal days (P) 41–43). BoNT/E solution (100 nM in phosphate-buffered saline (PBS, pH 7.4) containing 2% rat serum albumin) was injected stereotaxically at three locations (A = −2.0 mm, L = 1.0 mm; A = −4.0 mm, L = 1.0 mm; A = −6.0 mm, L = 1.0 mm) at a depth of 2 mm from the cortical surface (2 µl at each location; rate, 0.2 µl/min).

To label geniculocortical axons, we injected an anterograde tracer, biotinylated dextran amine (BDA, MW 10 kDa, Molecular Probe). A glass microelectrode filled with BDA solution (10% in 0.5 M NaCl) was positioned stereotaxically at LGN, which is ipsilateral to the BoNT/E injection sites, with verification of depth by monitoring visual responses. We used the pipettes with fine tips (5–10 µm diameter) to minimize tissue damage that might label the fiber of passage. BDA was injected iontophoretically (pipette positive current of 5 µA, 2 s ON/2 s OFF pulses, 60–70 times) at three to four sites in lamina A of LGN. A group of kittens (n = 3) was deprived of vision in both eyes by eyelid suture. All surgical procedures were performed under sterile condition under anesthesia with 2–3% isoflurane in N_2_O and O_2_ (1∶1). All the incisions were infiltrated with local anesthetics. The animals were administered an antimicrobial agent (enrofloxacin, 5 mg/kg) every day after the surgery until euthanasia.

### Histology

After a survival period of one week, the cortical region inactivated by BoNT/E was delineated physiologically by mapping cortical cell activity with a tungsten microelectrode placed at various distances from the sites of BoNT/E injection. The kittens were anesthetized with pentobarbital (Nembutal, Dainippon Sumitomo Pharma; initial dose, 30 mg/kg; maintenance dose, 2–4 mg/kg/hr) during recording experiments. When no spike activity except for injury discharges was recorded while the electrode was advanced to a depth of 2000 µm, we determined the site to be inactivated by BoNT/E. A 27 G needle was inserted to form marks at the border of the inactivated region. Then the animals were euthanized with an overdose of pentobarbital (100 mg/kg, i.v.) and perfused transcardially with cold PBS, followed by 4% paraformaldehyde in 0.1 M phosphate buffer (PB, pH 7.4). The brain was removed and postfixed in the fixative containing 20% sucrose. Tissue blocks containing LGN and the entire caudal pole of the cortex were cut on a freezing microtome in the frontal plane (50 µm thickness). All sections were collected in PBS and processed for the standard ABC method. Briefly, the sections were incubated overnight at 4°C in a blocking solution containing 5% bovine serum albumin (BSA), 3% normal goat serum (NGS) and 0.7% Triton X-100 in PBS. They were then transferred to a solution containing the avidin-horseradish peroxidase complex (Vector Laboratories, Burlingame, CA, USA), 0.1% Triton X-100 and 0.1% BSA in PBS and incubated overnight at 4°C. After washing in PBS and Tris-buffer (50 mM, pH 7.4), they were reacted with a solution containing 0.1% diaminobenzidine hydrochloride, 0.025% nickel ammonium sulfate, 0.025% cobalt chloride and 0.025% hydrogen peroxidase in Tris-buffer.

Selected sections containing the visual cortex were processed for immunohistochemical staining of Egr-1. The sections were incubated with the blocking solution overnight at 4°C. They were then transferred to a solution of 2.5% BSA and 5% NGS in PBS containing an Egr-1 antibody at a dilution of 1∶10000 (rabbit polyclonal antibody; Santa Cruz Biotechnology, Santa Cruz, CA, USA) and incubated overnight at 4°C. After washing in PBS and blocking, they were transferred to a solution containing 5% BSA, 2% NGS and 0.3% Triton X-100 in PBS containing a biotinylated secondary antibody (biotinylated goat anti-rabbit IgG; Vector Burlingame, CA, USA) and incubated for 1 hr at room temperature. After washing in PBS, they were transferred to a solution containing avidin-horseradish peroxidase and incubated overnight at 4°C. After washing in PBS and Tris-buffer, they were reacted with a solution containing 0.01% diaminobenzidine hydrochloride, 0.025% nickel ammonium sulfate, 0.025% cobalt chloride and 0.035% hydrogen peroxidase in Tris-buffer. All sections were mounted on MAS-coated slides, dehydrated in a graded series of ethyl alcohol, cleared in xylene, and coverslipped. Selected sections containing LGN or the visual cortex were stained with cresyl violet for localization of layer boundaries and injection sites.

### Axonal arbor analysis

All of the injection sites were well confined to a single lamina of LGN. When an injection site invades the other lamina representing the other eye, we eliminated all samples in the same animal from arbor reconstruction. We analyzed the axonal arbors in the area inactivated by BoNT/E defined physiologically. In addition, our analysis is limited to axons with well-filled arbors that could be followed up to their thinnest terminals. BDA-filled arbors were reconstructed at ×1000 magnification from serial sections in 3D with the aid of a computer graphic system (Neurolucida, Microbrightfield, VT). We evaluated the size and complexity of each arbor by measuring the total length of the arbor and the number of branch points, respectively. The total length of each arbor was obtained by the addition of lengths of all the branches constituting the terminal field of an arbor. Only the portion of the arbor located in layers II/III and IV was considered for these analyses. Very short endings less than 5 µm were omitted from the analyses.

### Western blotting

The adequate condition of BoNT/E injection was determined using rats. BoNT/E solution (50 nM in PBS containing 2% rat serum albumin) was injected at single location in area 17 of the visual cortex (A = 6.5 mm, L = 4.0 mm from bregma; depth from cortical surface, 0.8 mm) in 6-week-old rats using a 30 G stainless steel cannula (1.5 µl; rate, 0.05 µl/min). On various days after surgery, we recorded visual cortical activity with a tungsten microelectrode under anesthesia induced with pentobarbital (50 mg/kg, i.p.) After recording, the rats were euthanized with an overdose of pentobarbital (150 mg/kg, i.p.) and cortical tissue specimens including the visual cortex (A = 6.5–7.5 mm, L = 3.8–4.4 mm from bregma) were dissected out. The tissue specimens were homogenized with a glass-Teflon homogenizer and sonicated in RIPA buffer (150 mM NaCl, 1% NP-40, 0.5% deoxycholic acid, 0.1% SDS, 1 mM EGTA, 1 mM EDTA, 2 mM tetrasodium pyrophosphate, 4 mM para-nitrophenyl-phosphate, 1 mM sodium orthovanadate, 5 µg/mL aprotinin and 1 mM PMSF in 50 mM Tris-HCl, pH 7.4). The homogenates were centrifuged at 700 *g* for 10 min at 4°C. The resultant pellets were dissolved in the homogenizing buffer and stored at −80°C until western blotting was performed. Protein concentration was determined with a Micro BCA Protein Assay kit (Pierce, Rockford, IL, USA). Proteins in the samples were separated by 12% or 7.5% sodium dodecyl sulphate-polyacrylamide gel electrophoresis (SDS-PAGE), and transferred to a polyvinylidene difluoride membrane. The membranes were blocked with 5% skimmilk in Tris-buffered saline (TBS) containing Tween-20 (T-TBS) and incubated with T-TBS containing a primary antibody against SNAP-25 (1∶2000; mouse monoclonal antibody; Covance, Princeton, NJ, USA), Egr-1 (1∶2000) and glyceraldehyde 3-phosphate dehydrogenase (GAPDH; 1∶5000, mouse monoclonal antibody; Chemicon Temecula, CA, USA) overnight at 4°C. They were then incubated with a peroxidase-conjugated secondary antibody (1∶5000, anti-mouse IgG antibody, 1∶5000, anti-rabbit IgG antibody, 1∶10000, anti-mouse IgG antibody; GE Healthcare, Piscataway, NJ, USA) at room temperature for 1 hr. Signals were detected by enhanced chemiluminescence (ECL kit; GE Healthcare) and quantified using the ImageJ software (NIH, Bethesda, MD, USA). The blot densities of the proteins of interest were normalized to those of GAPDH in the same membrane.
